# Maternal burnout dynamics: identifying most vulnerable groups

**DOI:** 10.1192/j.eurpsy.2026.11664

**Published:** 2026-07-31

**Authors:** E. Polonetskaya, E. Pervichko

**Affiliations:** Faculty of Psychology, Lomonosov Moscow State University, Moscow, Russian Federation

## Abstract

**Introduction:**

While the first months of motherhood are traditionally considered to be the most challenging ones, it is essentially to scientifically research and understand the dynamics of the maternal burnous so the family members, the health professionals and the social services are warned accordingly.

**Objectives:**

Our research was aimed at studying the association between maternal burnout and the age of the mother, as well as burnout dynamics during the first years of motherhood.

**Methods:**

A maternal burnout questionnaire in Russian (the Maternal Burnout Assessment Tool, MBAT), being an adaptation of the Burnout Assessment Tool to the motherhood context, was used to assess burnout tendencies in mothers. The sample consisted of 792 Russian-speaking mothers with children up to seven years of age. Single-factor analysis of variance was used for data analysis (Kruskal-Wallis test).

**Results:**

As shown in Table 1 below, our research demonstrates that the highest level of the mean emotional impairment score (being one of the burnout symptoms) is associated with the youngest group of mothers. No further associations of the mother’s age with the burnout symptoms were discovered.

**Table 1.** Table 1. Long description.

Mother’s age	Younger than 25	From 25 to 30	From 31 to 35	From 36 to 40	Older than 40	*p*
**Emotional impairment**	3.07	2.78	2.94	2.99	2.79	0.015

As shown in Table 2 below by the mean scores, our research demonstrates that burnout symptoms accumulate and strengthen over time.

**Table 2.** Table 2. Long description.

Child’s age	Younger than 6 months	From 6 months to 1 year	From 1 year to 1 year and 6 months	From 1 year and 6 months to 3 years	From 3 years to 7 years	*p*
**Burnout**	2.51	2.53	2.27	2.77	2.77	<.001
**Exhaustion**	2.82	2.87	2.92	3.18	3.12	<.001
**Distancing**	1.98	1.93	2.07	2.21	2.18	0.007
**Emotional impairment**	2.84	2.83	2.79	3.08	3.02	0.005

**Conclusions:**

Based on the above, attention should be paid by the supporting caregivers and health professionals involved to the mothers mothers irrespective of their age and to mothers with children aged upwards of one and a half years as the most vulnerable group. Research on samples of mothers with schoolchildren and adult children is advisable.

**Disclosure of Interest:**

None Declared

## Long descriptions

### Table 1. Long description

The table consists of two rows and seven columns.

Row 1 (Header): Mother's age, Younger than 25, From 25 to 30, From 31 to 35, From 36 to 40, Older than 40, and p.

Row 2 (Data): Emotional impairment. The scores for each age group are as follows:

- Younger than 25: 3.07

- From 25 to 30: 2.78

- From 31 to 35: 2.94

- From 36 to 40: 2.99

- Older than 40: 2.79

- p-value: 0.015

Navigate back to Table 1..

### Table 2. Long description

The table consists of seven columns and five rows. The columns represent the child's age groups and a p-value column. The age groups are: Younger than 6 months, From 6 months to 1 year, From 1 year to 1 year and 6 months, From 1 year and 6 months to 3 years, and From 3 years to 7 years.

* Burnout: Scores are 2.51, 2.53, 2.27, 2.77, and 2.77 respectively, with a p-value less than .001.

* Exhaustion: Scores are 2.82, 2.87, 2.92, 3.18, and 3.12 respectively, with a p-value less than .001.

* Distancing: Scores are 1.98, 1.93, 2.07, 2.21, and 2.18 respectively, with a p-value of 0.007.

* Emotional impairment: Scores are 2.84, 2.83, 2.79, 3.08, and 3.02 respectively, with a p-value of 0.005.

Navigate back to Table 2..

